# Non-pharmacological labor pain relive methods: utilization and associated factors among midwives and maternity nurses in Najran, Saudi Arabia

**DOI:** 10.1186/s12978-023-01737-2

**Published:** 2024-01-24

**Authors:** Wafaa T. Elgzar, Majed S. Alshahrani, Heba A. Ibrahim

**Affiliations:** 1 Department of Maternity and Childhood Nursing, Nursing College Najran University, Najran, Kingdom of Saudi Arabia; 2 Department of Obstetrics and Gynecology, Faculty of Medicine, Najran University, Najran, Saudi Arabia

**Keywords:** Non-pharmacological methods, Labor pain, Associated factors, Nurses, Midwives

## Abstract

**Background:**

Traditionally, pharmacological pain relief methods have been the most acceptable option for controlling labor pain, accompanied by numerous adverse consequences. Non-pharmacological labor pain relive methods can reduce labor pain while maintaining an effective and satisfying delivery experience and delaying the use of pharmacological methods. This study explores the utilization of non-pharmacological labor pain relive methods and its associated factors among midwives and maternity nurses.

**Methods:**

A cross-sectional research was conducted in Maternal and Children Hospital/Najran, Saudi Arabia, from April to May 2023 and incorporated a convenience sample of 164 midwives and maternity nurses. The data was collected using a self-reported questionnaire composed of five sections; basic data, facility-related factors, non-pharmacological labor pain relive utilization and attitude scales, and knowledge quiz. A logistic regression was used to determine the associated factors with non-pharmacological labor pain relive utilization.

**Results:**

The results revealed that 68.3% of participants utilized non-pharmacological labor pain relive methods. The midwives and maternity nurses helped the parturient to tolerate labor pain by applying the non-pharmacological labor pain relive methods, including; positioning (55.5%), breathing exercises (53.7%), comfortable and relaxing environment (52.4%), therapeutic communication (47%), positive reinforcement (40.9%), relaxation (40.2%), and therapeutic touch (31%). In addition, working unit, providers-patient ratio, working hours, non-pharmacological labor pain relive training, years of experience, and non-pharmacological labor pain relive attitude were significant determinants of non-pharmacological labor pain relive utilization (P < 0.05).

**Conclusions:**

High non-pharmacological labor pain relive utilization was significantly associated with nurses' older age and higher education, working in the delivery room, lower nurse-patient ratio, lower working hours, in-services training, increased years of experience, and positive attitude. The study sheds light on the importance of handling the pre-mentioned factors to enhance non-pharmacological labor pain relive utilization.

## Background

Childbirth is a unique and multidimensional human experience associated with positive and negative feelings. Positive feeling includes excitement, hope, self-actualization, and celebration [1]. Negative feeling incorporates fear, anxiety, stress, insecurity, and expected pain [2]. Labor pain is an expected part of normal labor and is one of life's most memorable experiences. Labor pain has a visceral and somatic origin. The visceral pain occurred in the first stage of labor due to uterine contractions and cervical dilation [3]. The somatic pain occurred in the late first and second stages of labor and resulted from pressure excreted by the fetus’s head on the vagina and perineum [4]. Women’s ability to cope with labor pain is influenced by the pain threshold, fear, anxiety, culture, emotional and cognitive input, and progress of labor or direct sensory input [4, 5]. In addition, the neuromatrix theory of pain emphasizes the importance of any experience in pain perception and coping strategies [6].

Labor pain management is vital for parturient women and health care providers. Women’s satisfaction is mainly correlated with the quality of pain management. Although pain is not the only factor related to women’s satisfaction, it is crucial [7]. Pain management is a unique need for each woman, depending on her experience and physiological parameters. Leap et al. identified two main paradigms for pain management during labor: “pain relief” and “working with pain”. The pain relief paradigm is to get complete pain control during labor through the pharmacological option of management based on the belief that no women need to suffer during labor [8]. Complete pain relief is not always associated with higher birth satisfaction. According to Maimburg et al. women who received epidural anesthesia reported lower birth satisfaction after five years of follow-up assessment [9]. From a physiologic point of view, pharmacological pain management may be associated with numerous side effects and unfavorable outcomes such as nausea, vomiting, drowsiness [10], hypotension, headache, nerve injury, and urinary retention [11]. Kumar et al. reported that late preterm and full-term infants of mothers who received epidural anesthesia are more likely to develop neonatal respiratory distress [12]. Complications related to the course of labor may include delayed second stage, instrumental delivery, and cesarean section [10].

Working with pain paradigms is based on the concept that pain is a normal and necessary part of normal labor progress. Therefore, the role of health care providers is to encourage, help and advocate for women to deal with pain and emphasize the role of self-control and the ability to deal with stressful, painful conditions [13]. Consequently, the women may prefer to control pain through simple measures such as water immersion, guided imagery, changing position, breathing exercises, and other Non-pharmacological Labor Pain Relief (NPLPR) methods. According to previous systematic reviews, the NPLPR method promises a cost-effective, easily applicable, safe pain management option without complication to the woman, fetus, and labor progress. It also could reduce or delay the need for pharmacologic pain management [14, 15].

Midwives and maternity nurses are very important in women's care during labor. They can apply NPLPR methods; to improve the women's comfort, satisfaction, and the whole birthing experience [13]. In some instances, midwives and nurses may not apply NPLPR because of inadequate knowledge, negative attitude, decreased nurse-patient ratio, lack of experience, low education, inadequate training, and absence of NPLPR guidelines [16, 17]. No Saudi study in the international database evaluated the NPLPR utilization and its related factors. However, the availability of such data may pave the way to enhance NPLPR utilization through training, help, and follow-up. Therefore, the current study aims to explore the utilization of NPLPR methods and its associated factors among midwives and maternity nurses at the Maternity and Children Hospital, Najran, Saudi Arabia**.**

## Methods

### Study design and participants

A cross-sectional research design was conducted at maternity departments in Maternity and Children Hospital (MCH) /Najran, Saudi Arabia. Three departments were selected where normal labor may occur; the delivery room, emergency department, and inpatient maternity units, where some patients may be followed in the first stage of labor in case of a crowded delivery room. A convenience sample of all midwives and nurses working in the maternity departments and accepted to participate in the study were included. Midwives and nurses with less than one year of experience were excluded from the study.

### Data collection tools

The researchers developed a self-reported questionnaire based on the related recent literature [18, 19]. It encompasses five main sections; the first is basic data, the second is system or facility-related factors, the third is the NPLPR utilization scale, the fourth is the NPLPR attitude scale, and the fifth is the NPLPR knowledge quiz.

Basic data included age, religion, nationality, marital status, the highest level of education, and monthly income. System or facility-related factors to the utilization of NPLPR include working unit, profession, years of experience, providers: patient ratio, working hours, availability of NPLPR guidelines, and NPLPR training. The NPLPR utilization scale was developed to assess the frequency of utilization of different NPLPR modalities. It asses cognitive/behavioral (5 items), physical (9 items), emotional (2 items), environmental comfort (1 item), and patient/family involvement (3 items). The total scale composed of 20 items rated on a 5-point Likert scale ranged as always (5), often (4), sometimes (3), rarely (2), and never (1). The total scale score ranged from 20 to 100, and the participants were considered to have low (20–60) or high (61–100) utilization based on their total score. The NPLPR attitude scale is composed of 10 statements rated on a 5-point Likert scale ranging from strongly agree (5) to strongly disagree (1). The total scale score ranged from 10 to 50, the negative attitude from 10 to 23, neutral from 24 to 37, and the positive from 38 to 50. The NPLPR knowledge quiz: It was developed to evaluate the NPLPR definition, main types, benefits, and physiological background. The scale is composed of 8 dichotomous and multiple choice questions scored as the correct answer (2), incomplete answer (1), and incorrect answer (0). Poor knowledge is considered at 0–11, fair at 12–22, and good knowledge at 23–32.

The instrument was developed by the researchers; then, it was tested for face validity by a jury of 4 experts in obstetrics and gynecology nursing and a biostatistician. Tools' reliability was conducted with Cronbach’s Alpha test. The test results were 0.81 on the NPLPR utilization scale, 0.78 NPLPR attitude scale, and 0.77 on the NPLPR knowledge quiz.

### Sample size and sampling procedures

Epi Info free sample size calculator was used to calculate the sample size. The total number of midwives and nurses working in the delivery room, emergency department, and inpatient maternity units was 195 midwives and nurses. The parameters used for sample size calculation were 99.9% CI, 5% margin error, and a power of 99%. The prevalence of NPLPR utilization only or in combination with other pain relief medication was 78.4% from a prior study. They conducted a facility-based cross-sectional study to explore the attitude and utilization of NPLPR among obstetrics care providers in Ethiopia [20]. The calculated sample size was 152, and we added 15% for the anticipated nonresponse rate and the corrupted questionnaire. Therefore, the total sample size was 175. Convenience sampling was utilized until the desired sample size was reached. All the questionnaires were examined for data completeness, and 11 sheets were excluded. The final data analysis was done on 164 cases. In the case of the selected midwife/nurse who refused participation, she was replaced by another one.

Data collection started from April to May 2023. For better accessibility and collaboration of the nurses and midwives, one of them was recruited as a data collector. Before data collection, the research proposal, tools of data collection, and research ethics were explained to the data collectors. Then the self-reported questionnaire was distributed to the participants in paper form during their working hours.

### Ethical approval

Ethical approval was taken in four steps. Step 1: The research proposal was approved by the deanship of scientific research at Najran University. Step 2: The research proposal and questionnaire were approved by the ethical committee at Najran health affairs (IRB: 2023-06E). Step 3: Approval to start data collection was taken from the hospital administration. Step 4: Informed consent was written at the beginning of the questionnaire, and the participants were informed about their right to refuse participation without any penalties. Anonymity was applied, and all data was treated as confidential and used only for research purposes.

### Data analysis

The data was entered into SPSS version 23, and the necessary analysis was done. Descriptive statistics were used to describe data as numbers, percentages, mean, and standard deviations. Binary logistic regression was used to examine the predictors for high NPLPR utilization, and the Cox and Snell good of fitness test was used to examine the total model fit. The total knowledge, attitude, and utilization were calculated by summing items, and the significant level was considered at p < 0.05.

## Results

Table 1 shows that around one-half of the study, participants were Indian (51.2%) and Christian (51.8%). Besides, around three-quarters of the midwives and nurses were married (73.2%) and bachelor’s degree holders (75.0%). Nearly an equal proportion of the participants had not enough (47.6%) or just enough (46.3%) monthly income. The mean age of the study participants was 36.80 ± 8.97 years.

**Table 1:** Participants’ demographic data (n = 164)

Basic data	No (164)	%
Nationality		
Saudi	16	9.8
Egyptian	11	6.7
Sudanese	2	1.2
Filipino	51	31.1
Indian	84	51.2
Religion		
Muslim	48	29.3
Cristian	85	51.8
Hindu religion	24	14.6
Others	7	4.3
Marital status		
Single	41	25.0
Married	120	73.2
Divorced/widowed	4	1.8
Educational level		
High diploma	34	20.7
Bachelor’s degree	123	75.0
Master’s degree	7	4.3
Monthly income		
Not enough	78	47.6
Enough	76	46.3
Enough and can save	10	6.1
Age in years (mean ± SD)	36.80 ± 8.97	

Table 2 illustrates that 50.6% of the study participants were working in the inpatient maternity units, 87.8% were nurses, 56.1% had an undetermined provider-patient ratio, and 57.9% worked for 8 h daily. In addition, 59.1% received training about NPLPR during their formal education. All the participants (100.0%) reported the absence of any guidelines regarding NPLPR. The mean years of experience among the study participants were 10.77 ± 6.49.

**Table 2:** System or facility-related factors (n = 164)

System or facility-related factors	No	%
Working unit		
Emergency department	22	13.4
Delivery room	59	36.0
Inpatient maternity unit	83	50.6
Profession		
Midwife	20	12.2
Nurse	144	87.8
Providers: patient ratio		
1:4	28	17.1
1: 6	10	6.1
1: 8	34	20.7
Undetermined	92	56.1
Working hours		
8	95	57.9
12	54	32.9
More than 12	15	9.1
Presence of NPLPR guidelines		
Yes		
No	164	100
NPLPR training		
Never received	37	22.6
Yes, during my formal education	97	59.1
Yes, during my postgraduate education	9	5.5
Yes, training session after employment	21	12.8
Years of experience (mean ± SD)	10.77 ± 6.49	

Table 3 illustrates that NPLPR methods that don’t require equipment were most frequently applied, including positive reinforcement (40.9%) and relaxation (40.2%) under the cognitive NPLPR domain. The most frequently used modalities under the physical domain were positioning (55.5%) and breathing exercises (53.7%); however, trans-electrical nerve stimulation (11.6%) and Acupuncture/acupressure (11.0%) were the least frequently used modalities. Emotional modalities such as therapeutic communication (47%) and therapeutic touch (31%) were used at a high frequency because it requires no consent or equipment. Environmental comfort, as providing a comfortable and relaxing environment, was used among (52.4%). Besides, patient-family involvement, including providing education for patients and families (50.0%), counseling (46.3%), and educating the patient about bearing down (42.1%), were highly utilized.

**Table 3:** NPLPR methods utilization among midwives and maternity nurses (n = 164)

	Always		Often		Sometimes		Rarely		Never used	
	No.	%	No.	%	No.	%	No.	%	No.	%
Co-cognitive-behavioral										
Guided imagery	44	26.8	51	31.1	34	20.7	17	10.4	18	11.0
Relaxation	66	40.2	56	34.1	29	17.7	7	4.3	6	3.7
Positive reinforcement	67	40.9	58	35.4	28	17.1	8	4.9	3	1.8
Distraction	48	29.3	53	32.3	37	22.6	17	10.4	9	5.5
Virtual reality	19	11.6	18	11.0	33	20.1	39	23.8	55	33.5
Physical										
Thermal stimulation (cold or hot)	51	31.1	49	29.9	35	21.3	15	9.1	14	8.5
Trans electrical nerve stimulation	19	11.6	26	15.9	32	19.5	43	26.2	44	26.8
Massage	48	29.3	49	29.9	33	20.1	16	9.8	18	11.0
Breathing technique	88	53.7	38	23.2	27	16.5	8	4.9	3	1.8
Positioning	91	55.5	42	25.6	27	16.5	3	1.8	1	0.6
Hydrotherapy/patient bathing	40	24.4	51	31.1	27	16.5	13	7.9	33	20.1
Resting	81	49.4	44	26.8	24	14.6	9	5.5	6	3.7
Acupuncture/acupressure	18	11.0	51	31.1	26	15.9	14	8.5	55	33.5
Herbal drink	32	19.5	45	27.4	29	17.7	16	9.8	42	25.6
Emotional										
Therapeutic touch	51	31.1	53	32.3	28	17.1	15	9.1	17	10.4
Therapeutic communication	77	47.0	37	22.6	25	15.2	15	9.1	10	6.1
Environmental comfort (providing a comfortable, relaxing environment)	86	52.4	39	23.8	21	12.8	12	7.3	6	3.7
Patient-family involvement										
Counseling	76	46.3	40	24.4	30	18.3	13	7.9	5	3.0
Providing education for patients and families	82	50.0	38	23.2	24	14.6	14	8.5	6	3.7
Educate the patient about bearing down	69	42.1	45	27.4	29	17.7	13	7.9	8	4.9

Table 4 summarize that 76.8% and 81.1% of the participants had good knowledge and positive attitude regarding NPLPR, respectively. In addition, 68.3% of the participants reported high utilization of NPLPR methods.

**Table 4:** NPLPR total knowledge, attitude, and utilization among midwives and maternity nurses (n = 164)

Variables	No.	%
Total knowledge		
Poor	5	3.0
Fair	33	20.1
Good	126	76.8
Mean ± SD	13.46 ± 2.73	
Total attitude		
Negative	4	2.4
Neutral	27	16.5
Positive	133	81.1
Mean ± SD	40.88 ± 5.74	
Total utilization		
Low	52	31.7
High	112	68.3
Mean ± SD	70.56 ± 17.30	

Table 5 illustrates that level of education and age were positive demographic predictors for high NPLPR utilization. A midwife/nurse with a master’s education had a 3.3 higher probability of utilizing NPLPR methods when compared with a high diploma midwife/nurse [3.313 (0.978–11.125), p = 0.043]. Besides, an increase of one year in the midwife/nurse’s age increased her probability to practice NPLPR 1.7 times [1.780 (1.051–1.872), p = 0.035).

**Table 5:** Demographic predictors of high NPLPR utilization

Demographic data	AOR (95% CI)	p
Nationality		0.231
Saudi	Refs.	
Egyptian	1.102 (0.481–2.678)	0.785
Sudanese	1.663 (0.572–4.931)	0.366
Filipino	0.391 (0.088–1.721)	0.221
Indian	1.171 (0.479–2.831)	0.730
Religion		0.189
Muslim	Refs.	
Cristian	1.243 (0.379–4.076)	0.719
Hindu religion	0.871 (0.341–1.962)	0.675
Indian	0.662 (0.080–5.467)	.702
Marital status		0.825
Single	Refs.	
Married	0.930 (0.372–2.380)	0.782
Divorced/widowed	1.117 (0.385–3.171)	0.841
Educational level		0.016^*^
High diploma	Refs.	
Bachelor’s degree	1.248 (0.467–3.352)	0.557
Master’s degree	3.313 (0.978–11.125)	0.043^*^
Monthly income		0.910
Not enough	Refs.	
Enough	0.881 (0.391–1.984)	0.759
Enough and can save	0.729 (0.140–3.800)	0.708
Age in years (mean ± SD)	1.780 (1.051–1.872)	0.035^*^
− 2 Log likelihood (187.513)	Cox & Snell R Square (0.042)	Nagelkerke R Square (0.061)

AOR: Adjusted Odd Ratio CI: Confidence Interval

*Significant at p < 0.05

Table 6 shows that working unit, providers: patient ratio, working hours, NPLPR training, years of experience, and NPLPR attitude were predictors for NPLPR utilization. Working in a delivery room [AOR = 1.631(0.741–3.868), p = 0.039] or inpatient maternity units [AOR = 1.671(1.057–4.021), P = 0.048] increased the probability for higher NPLPR utilization by1.6 times when taking emergency department as a reference. Receiving NPLPR training during formal education increased the probability of higher NPLPR utilization four times compared with midwife/nurse who received no training [AOR = 4.191(1.583–11.094), p = 0.004]. In addition, one year increase in working experiences increased the nurse probability for higher NPLPR practices by six times [AOR = 6.501(1.012–41.764), p = 0.049). Also, an increase of one point in the participants' NPLPR attitudes increased the probability of higher application of NPLPR by one time [AOR = 1.125(1.013–1.249), P = 0.028]. On the other hand, provider: patient ratio of 1:6 [AOR = 0.165 (0.055–0.432), p = 0.000] or 1:8 [AOR = 0.155 (0.046–0.531), P = 0.003] decreased the midwife/nurse probability to provide NPLPR when compared to a ratio of 1:4. Furthermore, working for 12 [AOR = 0.712 (0.242–2.872), p = 0.048] hours or more than 12 h [AOR = 0.205 (0.061–0.712), p = 0.013] decreased the midwife/nurse probability to utilize NPLPR when taking working for 8 h as a reference.

**Table 6:** System or facility-related predictors of high NPLPR utilization

Facility-related predictors	AOR (95% CI)	p
Working unit		0.018^*^
Emergency department	Refs.	
Delivery room	1.631 (0.741–3.868)	0.039*
Inpatient maternity units	1.671 (1.057–4.021)	0.048*
Profession		
Midwives	Refs.	
Nurse	1.182 (0.798–1.872)	0.424
Providers: patient ratio		0.001^*^
1:4	Ref	
1: 6	0.165 (0.0550.432)	0.000^**^
1: 8	0.155 (0.046–0.531)	0.003^*^
Undetermined	0.765 (0.337–1.629)	0.467
Working hours		0.000^**^
8	Refs.	
12	0.712 (0.242–2.872)	0.048^*^
More than 12	0.205 (0.061–0.712)	0.013^*^
Training related to the utilization of non-pharmacological pain relief		0.036
Never received		Ref
Yes, during my formal education	4.191 (1.583–11.094)	0.004^**^
Yes, during my postgraduate education	3.139 (0.515–19.140)	0.215
Yes, training session after employment	3.170 (0.803–12.504)	0.099
Years of experience (mean ± SD)	6.501 (1.012–41.764)	0.049^*^
Total knowledge	0.949 (0.781–1.153)	0.598
Total Attitude	1.125 (1.013–1.249)	0.028^*^
− 2 Log likelihood (279.258)	Cox and Snell R Square (0.487)	Nagelkerke R Square (0.061)

AOR: Adjusted Odd Ratio CI: Confidence Interval

^*^Significant at p < 0.05

^**^Significant at p < 0.001

## Discussion

Traditionally, pharmacological pain relief methods have been the most acceptable option for controlling labor pain, accompanied by numerous adverse consequences [21]. NPLPR methods can reduce labor pain while maintaining an effective and satisfying delivery experience and reducing obstetrics interventions [22]. However, no research concerning the utilization of NPLPR in Saudi Arabia has been found in international data based.

The result of the present study disclosed that more than two-thirds of the participants had a high utilization of NPLPR. This finding aligns with the study conducted in Nigeria, which stated that 65.2% of nurses used non-pharmacological techniques to manage patients’ pain [23]. Another Turkish study assessed the nurse’s knowledge and practices regarding non-pharmacological pain relief practice and revealed that 62.4% of their participants utilized a non-pharmacological approach in their daily practice [24]. However, our result is higher than Eyeberu et al.; Getu et al.; and Bishaw et al. studies conducted in different health facilities in Ethiopia, 59.3%, 46.8%, and 34.4%, respectively [20, 25, 26]. Besides, a cross-sectional study conducted in Egypt to evaluate critical care nurses’ utilization of non-pharmacological pain relief options showed that 32.7% of nurses’ had satisfactory practices [17]. These discrepancies can be attributed to the differences in the non-pharmacological pain relief utilization scale. Our scale included different modalities, such as counseling and education provided for patients and families, which are conducted as a part of patient care protocol and informed consent in MCH. At the same time, the latter group studies which reported lower NPLPR utilization did not include these modalities in their scales, in addition to the obvious differences in the level of knowledge and attitude towards non-pharmacological pain relief.

The current study indicated the utilization of relaxation, positive reinforcement, breathing exercises, positioning, therapeutic touch, and therapeutic communication. In addition, to the environmental comfort (providing a comfortable and relaxing environment), counseling, providing education for patients and families, and educating the patient about bearing down were highly utilized at a high frequency. These findings are similar to what was reported in a Tanzanian study, where the majority of nurse-midwives reported utilization of various NPLPR modalities, including emotional support, deep breathing exercises, and position change during the first stage of labor [27]. Also, reassurance, psychological support, lower back massage, and breathing exercises were reported in prior studies in Ghana [28, 29].

A higher NPLPR utilization was associated with some demographic characteristics, such as midwives’/nurses’ level of education and age. These findings agree with the study by Olmstead et al. [30] who reported that older nurses were more likely to utilize non-pharmacological pain relief methods as a distraction to mitigate the suffering of children during procedural pain than younger nurses. In addition, other previous studies in Nigeria [23], Saudi Arabia [31], Eritrea [32], indicated that an increase in age and higher educational level of nurses were positive predictors of high non-pharmacological pain relief practice.

The present study results pointed out that NPLPR training is one of the facility-related factors affecting NPLPR utilization among midwives and maternity nurses. Receiving NPLPR training during formal education increased the likelihood of using the NPLPR four times higher than nurses without training. Several prior studies conducted in Saudi Arabia [31], Turkey [33], and Ethiopia [34] indicated that in-service training was an important factor associated with non-pharmacological pain relief practices. Therefore, the current study sheds light on the important role of in-service NPLPR training in improving midwives’ and maternity nurses’ awareness and enhancing their attitudes toward its application.

Moreover, in the current study, the utilization of NPLPR was associated with working experience. This could be attributed to the fact that those serving longer had acquired sufficient knowledge, expertise, and competence to efficiently manage labor using NPLPR. In this regard, the study conducted by Aschenbrenner assessed the association between work experience in childbirth units and their labor support attitudes. They found that nurses with more clinical experience had a greater impact on their attitudes and intention to provide labor pain management, leading to increased use of NPLPR [35]. Also, much evidence confirmed the association between nurses’ working experience and non-pharmacological pain relief utilization [20, 30, 32, 36].

The results of our study reported that the low utilization of NPLPR was attributed to an increased patient-provider ratio and working hours. Similar findings are found in the study conducted by Aziato et al. [28] which found an association between inadequate non-pharmacological pain relief utilization and the nurses' heavy workload, which prevented them from providing adequate care. Hildingsson et al. also found that a shortage of staff, inadequate resources, and stressful work environments were associated with experience burnout among midwives [37]. The shortage of staff nurses and midwives presents a challenge that policymakers should address. The Saudi Ministry of Health should ensure enough staff in labor units to create a conducive working environment for nurses and midwives and consequently encourage NPLPR utilization.

In the current study, an increase of one point in the participants’ NPLPR attitudes increased the probability of higher application of NPLPR by one time. This implies that nurses’ favorable attitudes toward NPLPR strategies facilitate their higher application in clinical practice. Our finding agrees with the studies in Ethiopia [34, 36, 38] and Nigeria [23]. All of them found that a positive attitude had significantly associated with higher non-pharmacological pain relief practices. Therefore, promoting the nurse’s attitude toward the impact of NPLPR methods is crucial. The current study provided essential data regarding NPLPR utilization in Najran, Saudi Arabia. The data provided by the current study clarified the predictors for NPLPR utilization, which should be considered in in-service training for midwives and nurses.

### Study strengths and limitations

This is the first study in Saudi Arabia that aimed to explore the utilization of NPLPR methods and it is associated factors among midwives and maternity nurses. However, due to the study’s cross-sectional design, it is challenging to establish a cause-effect relationship between the dependent and independent variables. Additionally, self-reported measures were used in our research methods, which may be influenced by individuals' memory and recall bias.

## Conclusion

Approximately two-thirds of midwives and maternity nurses highly utilized NPLPR methods. The most popular methods were relaxation, positive reinforcement, breathing exercises, positioning, therapeutic touch, therapeutic communication, comfortable and relaxing environment, counseling, and education for patients and families. Significant predictors for higher NPLPR utilization were nurses’ older age and high educational level, work in the delivery room, lower provider: patient ratio, lower work hours, NPLPR training, increased years of experience, and positive NPLPR attitude. The study sheds light on the important role of the pre-mentioned factors, especially the in-service training, in enhancing NPLPR utilization and continuation among midwives and maternity nurses.

## Data Availability

The datasets used during the current investigation are available from the corresponding author on reasonable request.

## Abbreviations

NPLPR: Non-pharmacological labor pain relive
MCH: Maternal and children hospital
AOR: Adjusted Odd Ratio
CI: Confidence Interval
